# Care relationships at stake? Home healthcare professionals’ experiences with digital medicine dispensers – a qualitative study

**DOI:** 10.1186/s12913-018-2835-1

**Published:** 2018-01-15

**Authors:** Sigrid Nakrem, Marit Solbjør, Ida Nilstad Pettersen, Hanne Hestvik Kleiven

**Affiliations:** 1 Department of Public Health and Nursing, Faculty of Medicine and Health Science, NTNU Norwegian University of Science and Technology, Trondheim, Norway; 2 Department of Design, Faculty of Architecture and Design, NTNU Norwegian University of Science and Technology, Trondheim, Norway; 3 Queen Maud University College, Trondheim, Norway

**Keywords:** eHealth, Empowerment, Healthcare professionals, Health technology, Home healthcare, Implementation, Independent living, Innovation, Qualitative method, Remote surveillance

## Abstract

**Background:**

Although digital technologies can mitigate the burdens of home healthcare services caused by an ageing population that lives at home longer with complex health problems, research on the impacts and consequences of digitalised remote communication between patients and caregivers is lacking. The present study explores how home healthcare professionals had experienced the introduction of digital medicine dispensers and their influence on patient-caregiver relationships.

**Methods:**

The multi-case study comprised semi-structured interviews with 21 healthcare professionals whose home healthcare service involved using the digital medicine dispensers. The constant comparative method was used for data analyses.

**Results:**

Altogether, interviewed healthcare professionals reported three main technology-related impacts upon their patient-caregiver relationships. First, national and local pressure to increase efficiency had troubled their relationships with patients who suspected that municipalities have sought to lower costs by reducing and digitalising services. Participants reported having to consider such worries when introducing technologies into their services. Second, participants reported a shift towards empowering patients. Digital technology can empower patients who value their independence, whereas safety is more important for other patients. Healthcare professionals needed to ensure that replacing care tasks with technology implies safe and improved care. Third, the safety and quality of digital healthcare services continues to depend upon surveillance and control mechanisms that compensate for less face-to-face monitoring. Participants did not consider the possibility that surveillance exposes information about patients’ everyday lives to be problematic, but to constitute opportunities for adjusting services to meet patients’ needs.

**Conclusions:**

Technologies such as digital medicine dispensers can improve the efficiency of healthcare services and enhance patients’ independence when introduced in a way that empowers patients as well as safeguards trust and service quality. Conversely, the patient-caregiver relationship can suffer if the technology does not meet patients’ needs and fails to offer safe and trustworthy services. Upon introducing technology, home healthcare professionals therefore need to carefully consider the benefits and possible disadvantages of the technology. Ethical implications for both individuals and societies need to be further discussed.

## Introduction

Demographic changes towards an ageing population that lives at home for longer with complex health problems have increased the need for healthcare services [1]. In Norway, national health reforms have increasingly shifted responsibility for long-term care recipients to municipal health authorities [2]. Today, long-term care is established as a public service in all Norwegian municipalities, and comprises home healthcare (24 h services), residential facilities and nursing homes for short-term and long-term care [2, 3]. Such expansion has increasingly burdened municipal economies, and, over time, might prove unsustainable. To address those challenges, proposed solutions have identified a range of innovations that can maximize the quality and efficiency of care [4].

Home healthcare services involve a multidisciplinary workforce, including both general practitioners (GPs) and healthcare professionals with bachelor’s degrees such as registered nurses (RNs), occupational therapists and physiotherapists. In addition, homecare services employ accredited social educators (ASEs), who have bachelor’s degrees in care for people with intellectual disabilities or dementia, as well as nurse assistants who perform tasks related to medication. The responsibilities of home healthcare services range from practical assistance with patients’ households and day-to-day life to advanced medical treatment involving medication. Patients who receive healthcare services at home vary considerably in function, age, and living conditions, as well as in terms of their illnesses and diseases [2]. As solutions to meet demands of municipal healthcare services, telecare and assistive technologies have been introduced, including digital medicine dispensers with remote follow-up, all of which offer promising opportunities [5–7]. However, given the relational nature of healthcare, face-to-face interaction between healthcare professionals and patients is considered essential [8]. Remote or technology-mediated care is often liable to pose new challenges and generate novel ethical implications. In response, the study presented here sought to illuminate relational aspects of healthcare related to the implementation of digital assistive technology devices for medication in home healthcare services.

## Background

Among older people, the intake and administration of medicines has high occurrence of errors [9–11], is a major cause of hospital admissions [12], and increase the risk for death [13]. In Norway, if healthcare services are responsible for assisting patients living at home with medication, national regulations require RNs and ASEs to manage all medication-related services [14], including administrating medication, reminding patients to take correct doses of medicine at certain times, contacting GPs to refill prescriptions, and helping patients to purchase medicine at pharmacies [15]. However, since healthcare professionals often visit patients several times a day to administer prescription medications, medication assistance often consumes a great deal of their daily schedules [16]. Alternatively, machine-based reminder and support systems are promising means to effectively follow up on a patient’s intake of medication and other medical procedures that patients perform themselves [10, 17, 18]. Although studies have indicated that using digital monitoring and digital pill dispensers increases adherence to treatment [19, 20], research that focuses on the use of digital medicine dispensers as part of home healthcare remains scarce, as does research on the general role of such assistive technology in healthcare services.

Complex and contextual healthcare depends heavily on the relationship of the healthcare professional and the patient, which, unlike other social relationships, is marked by exchange of sensitive personal information in a professional style of communication [21]. The caring relationship is one that affects both the person cared-for and the caregiver [22]. For that relationship to succeed, not only does the healthcare professional need communication skills, but the relationship itself requires a significant level of trust between the professional and the patient [23]. In long-term healthcare at home in particular, since patients may need to receive care several times each day and therefore meet many different professionals, trust and continuity in patient-caregiver relationships can be challenging to achieve [24]. Typically, face-to-face interaction and physical presence enable healthcare professionals to observe patients [25], who in turn, forge relationships with those professionals not only during verbal and non-verbal communication, but also by experiencing the performance of care [26, 27]. Digitalisation and remote healthcare can inhibit the development of trusting relationships because they alter the style of communication between patients and healthcare professionals. As a result, being physically distant in patient contact can demand new strategies for developing good relationships and trust. As studies have shown, for example, for telecare nurses to maintain good patient-caregiver relationships, when they are not meeting with patients face-to-face, they need to confirm that both they and their patients can trust the technology that they use [23, 28].

Of course, the use of technology in healthcare is nothing new for healthcare professionals. They have long applied a range of technologies to assist themselves and patients [8, 29]. However, today medical technologies increasingly involve digital components, pose new opportunities for Internet-based remote monitoring and exploit mobile technology and smartphones. As research on information and communication technology as support in home healthcare services has shown, such technology presents two main challenges: user-friendliness, and the clinical appropriateness of technology [30]. Beyond that, knowledge on the impact and consequences of implementing new technology in connection with home healthcare services remains scarce [31]. In response, we examined healthcare professionals’ experiences with digital medication technology, particularly its effects on the relational aspects of care in home healthcare services.

## Aim of the study

The aim of this study was to explore how healthcare professionals have experienced the introduction of digital medicine dispensers in home healthcare services and their influence on patient-caregiver relationships.

## Methods

Our qualitative multi-case study [32] involved semi-structured group and individual interviews with healthcare professionals whose work involves using digital medicine dispensers in home healthcare services. We used a qualitative design since this is considered particularly valuable for exploring areas not widely studied [33]. The aim was to gain insights into the healthcare professionals’ experiences with digital medicine dispensers and how the technology has influenced their relationship with home healthcare patients by examining diverse perspectives on delivering home healthcare services [34, 35].

### Setting and technology

The study was conducted in five Norwegian municipalities that had piloted the use of digital medicine dispensers in their home healthcare services, a small rural municipality (< 5.000 inhabitants; Case A), two mid-sized municipalities with mixed urban and rural areas (5.000–49.999 inhabitants; Cases B and C), and two larger municipalities with densely populated urban areas (> 50.000 inhabitants; Cases D and E). During the pilot period, each municipality introduced digital medicine dispensers into its home health services for 3–20 users. In most cases, different digital medication products had been tested, and healthcare professionals had contributed to product development by providing product suppliers with feedback based on their experiences.

Four of the five municipalities opted to test a portable carousel-type dispenser that nurses filled with tablets once per week. When it was time to take a certain medication, the dispenser sounded an alarm and transported a dose of the medication to a slot from where the patient could fetch it. By contrast, one municipality used a machine that transported a roll of pre-filled multi-dose bags, prepared by a pharmaceutical company, with all medicines for each dose in a separate bag. For each patient, a nurse (RN or ASE) loaded a roll of bags into the machine, which was programmed to sound an alarm when the patient needed to take medication. At the cue of the alarm, the patient pushed a button on the machine to release a bag, which the patient tore from the roll to obtain the medicine. In all cases, the digital medicine dispenser systems were equipped with telecare opportunities to notify the home healthcare service or relatives, in most cases via mobile phone, if a patient failed to take the medicine.

### Recruitment and sample

A purposive sample of cases and healthcare professionals was recruited, since we specifically wanted participants who had been using digital medicine dispensers, or were responsible for programming dispensers or refilling them in the patients’ homes. The home healthcare director or local project manager in each of the five municipalities asked healthcare professionals with such experience to participate, and gave the interviewer contact information of those who consented to participate. The interviewer contacted each professional and scheduled an individual interview with him or her to occur at his or her workplace or by telephone. The three pharmacists were interviewed together as a group. Table 1 provides an overview of all 21 participants, whose anonymity we have ensured by not reporting their age, gender and work tenure.

**Table 1:** Participants

Municipality/organization	Participants
A. Small municipality	Registered Nurse Licensed Practical Nurse Pharmacists (group of 3) Nurse Manager General Practitioner
B. Medium sized municipality	Occupational Therapist Registered Nurse/Manager Licensed Practical Nurse
C. Medium sized municipality	Occupational Therapist Registered Nurse Licensed Practical Nurse Registered Nurse/Manager
D. Large municipality	Registered Nurse/Project manager Registered Nurse/Manager Licensed Practical Nurse Registered Nurse/Manager
E. Large municipality	Occupational Therapist Physiotherapist Licensed Practical Nurse Physiotherapist/Project Manager
F. Company/technology supplier	Registered Nurse/training representative

### Data collection

Guided by our previous research and literature, we developed a thematic interview guide addressing three major topics with additional open-ended questions and probes [36], as detailed in Table 2. Interviews were conducted by two researchers from the research team during March 2014–January 2015. For practical reasons, six individual interviews occurred over the phone, whereas all other individual interviews and the group interview were conducted in person in a meeting room at each participant’s work-place. Each interview lasted approximately 1 h and was recorded and transcribed verbatim. Quotations from interviews presented here have been modified from oral expressions to written language in order to promote their comprehensibility. When preparing the manuscript, those quotations were translated from Norwegian to English by a native English speaker not on the research team.

**Table 2:** Interview guide

Topics
1. Changes in the relationship between professions and in professional roles
2. Innovation and change management in the home healthcare service and what facilitates and hinders technology implementation
3. The home as a place to work, related to issues of care and patient empowerment

### Data analyses

The constant comparative method was used to analyse the transcribed interviews [34]. This facilitated the identification of themes and possible analysis of differences between individuals (i.e. healthcare professionals) and cases (i.e. municipalities). All authors read all transcripts several times to get an overview of possible themes and ultimately we identified three primary themes that also reflected the three major topics in the interview guide: ‘*Professional roles and cooperation’, ‘Innovations and change management’, and ‘The home, and healthcare professionals’ experiences of the technology’*. For the present article, we analysed data from the first and third theme, whereas data regarding the second theme will be reported elsewhere.

During analyses of data concerning the two themes, all authors participated in coding interviews in detail, with special attention to differences and similarities among cases and professional groups. The research team held several meetings to discuss codes and their content, during which different interpretations were deliberated and reinterpreted, until consensus of interpretation was reached. The first and second author coded all interviews, and each code was subject to categorisation of meaning. By comparing codes (finding similarities) and contrasting codes (searching for negative cases), the final three analytical categories related to healthcare professionals’ experiences with relational aspects of care emerged.

### Ethics

The study was approved by the Norwegian Centre for Research Data - NSD (reference no. 37655). Each participant signed a written form of consent after receiving oral and written information about the study. Since interviews conducted at work places could have enabled participants to identify each other, all identifiable characteristics are excluded from the presentation of data here to ensure the anonymity of all individuals.

## Results

All participants reported that using digital medicine dispensers influenced their relationships with patients in terms of personal interaction and healthcare delivery. Since no major differences regarding reported experiences among health professionals and municipalities appeared, we present our results without differentiating groups of participants. From the interviews, three analytical categories associated with changes in caregiver-patient relationships related to using digital medicine dispensers in home health services emerged: 1) *National and local pressure to make services more efficient*; 2) *Shifts towards empowering patients*; and 3) *Surveillance mechanisms in the technology*.

### National and local pressure to make services more efficient

Participants reported that pressure from national and local authorities to maximise efficiency was a chief driver for implementing digital medicine dispensers in home healthcare services. For home healthcare professionals, pressure to free up time and resources had influenced their relationships with patients. They had already faced time constraints in delivering adequate care, and recognised that traditional face-to-face care would become unsustainable. Short visits for medication assistance were unduly expensive since travel time was the same regardless of the visit’s purpose. The home healthcare professionals expressed that leaving medication to digital medicine dispensers afforded them more time to deliver quality care to patients with greater needs: ‘*We can manage our time better. Instead of coming and going all of the time, it is better if we sit down and spend some time with the user.’* (Nurse).

To encourage patients to trust the technology, home healthcare professionals also expressed the importance of their confidence that the technology’s benefits outweighed its potential disadvantages. For some professionals, such confidence was necessary to successfully implement the technology. Some home healthcare professionals reported troubled relationships with patients who feared reduced services upon consenting to use the medicine dispenser. In introducing the technology to patients, the professionals had to consider such worries:

*My impression is that many older users keep wondering what the municipality will save money on this time. They often say ‘I want to have what I’ve been promised. What are you going to deny me today?’. So* [it is important to make clear] *that we work to achieve the same goal and to tell them that I’m not here to deny them a lot of things and withdraw the healthcare that you should have. (Occupational therapist)*

However, participants also reported that pressure to be more efficient benefitted patients as well. In general, assistive technology could allow older patients to grow old in their homes, thereby delaying their institutionalisation and supporting their autonomy, which the home health professionals identified as an important message to communicate to patients in order to maintain trust in their relationships. Offering a medicine dispenser instead of time-consuming traditional medication procedures could also mean higher-quality care and safety in medication therapy: ‘*Cost savings becomes a side effect. In my view,* it [the medicine dispenser] *improves the quality of services and makes them safer’* (Nurse).

### Shifts towards empowering patients

Participants reported that another major driver of implementing digital technology in home healthcare services was its contribution to empowering the technology’s users (i.e. patients). Whereas traditional healthcare can be paternalistic and create passive, dependent patients, the new technology can support patient autonomy, at least in parts of care, as home healthcare professionals expressed, in particular adding that the traditional healthcare model, with its frequent home visits could strain some patients. As part of that model, medication assistance often consists of brief, hurried, and often delayed visits from nurses that patients have to stay at home to receive, as one nurse explained: ‘*They* [patients] *need to wait for home healthcare services every time, which is exhausting and also ties them down.*

Home healthcare professionals reported that both younger and older home healthcare service users wished to be independent. For many participants, the context for their positive experiences with digital medicine dispensers was the introduction of a national healthcare model called ‘Everyday Rehabilitation’, the healthcare philosophy of which prioritises helping patients to cope with chronic disease and supporting active living. Home healthcare professionals hoped that assistive technology could be part of their efforts to achieve that goal for the sake of patients as individuals. As an occupational therapist explained:

*Everyday rehabilitation becomes increasingly important to integrate welfare technology into the daily lives of users. I always like to stress that. As an occupational therapist, my experience with assistive tools is that it’s irrelevant how many gadgets are available. What counts is that the assistive technology makes users more active. If it makes users more passive instead, then we have failed.*

However, a reported disadvantage of the technology was that care focused on empowerment and self-care has not been suitable for all, especially not frail and dependent older patients. Participants agreed that many patients continue to need so-called ‘traditional care’, involving professional’s presence in order to ensure that adequate healthcare is provided. Regarding such users, participants expressed that patient-caregiver relationships would suffer if care were administrated by way of remote technology. Healthcare professionals remained wary about which users should be equipped with digital medicine dispensers, and, in particular, when during the course of care such interventions were appropriate:

*I think it comes down to* *when* *we start using dispensers for our users. It’s important that they’re not too ill at that point. In one case we responded too late. She* [the user] *couldn’t handle using it* [the dispenser] *and unplugged it. In that case we should have intervened earlier, before her dementia had progressed too far* (Nurse assistant).

Of course, no participant characterised medication assistance technology as the sole point of contact with patients, but instead conceived the technology as a means to enhance care. Many participants reported that successfully implementing digital medicine dispensers into care required good patient-caregiver relationships, which themselves required building trusting relationships by ways of face-to-face interaction and by assessing individual care needs before the introducing of technology. In any case, information about the technology had to be modified to suit each individual patient and his or her situation.

Lastly, whereas advocating independence was convincing for some patients, advocating safety was more convincing for others. Although participants stated that taking the time to educate patients was important, they added that healthcare professionals also need to ensure that delegating healthcare tasks to technology devices would result in safe, improved care. Professional experiences had taught them that healthcare professionals’ insecurity with new technology would negatively affect their relationships with patients, who would develop the same sort of insecurity. As a nurse working for a technology supplier summarised: ‘*Insufficient knowledge makes people “rush” things. Health professionals aren’t trained, and users don’t get sufficient instructions and become anxious.’*

### Surveillance mechanisms in the technology

Participants reported that when healthcare professionals think that technology can improve quality and safety in care, they express a positive attitude towards the technology. They added that safety and quality can benefit from control mechanisms that compensate for fewer face-to-face services, including alarms that notify the home healthcare service. Surveillance mechanisms in medicine dispensers can also provide knowledge about the medication habits of patients who declined to receive visits at home from home healthcare services:

*We* [the home healthcare service providers] *have a woman with diabetes who’s been badly regulated. We think it’s partially because she forgets to take her medication, since we’ve found three or four doses left when we replaced her pill dispenser. She hasn’t requested more follow-up visits from home healthcare services, and we replace her pill container only once a week. We’ve discussed the situation with her several times, but nothing happened.* [With the new digital dispenser], I now *hope to see a long-term improvement in her blood sugar level* (Nurse).

However, participants also expressed that surveillance sometimes monitors not only medication practices, but also aspects of users’ lifestyles. Even if home healthcare professionals had programmed digital dispensers in cooperation with users, aspects of users’ habits became apparent because surveillance allowed insights into their lifestyles at home, as a nurse recalled to a particular patient:

*I had a patient. We* [She and I] *agreed that the dispenser could be left open from 8 to 9 in the morning. Great, I thought, it’s programmed accordingly. But then the alarm went off, several days in a row. I soon realised that she doesn’t wake up early, yet wished to give the impression that she does, because she thinks it’s embarrassing to not be an early riser. We* [the healthcare professionals] *can see what sort of lives patens lead, and their true habits become disclosed.*

Participants explained that they often discuss with each other the ethical challenges of their relationships with patients when delivering digital health services. The most common challenge related to the dichotomy of warm hands and cold technology - warm hands being synonymous with compassionate care delivered individually and cold technology being associated with technical surveillance and control of patients’ lives. Although reducing care to rote technical tasks concerned participants, possibilities for control and surveillance were not conceived as problematic, but advantageous for relationships with patients since such means can better inform healthcare professionals and help them to accommodate patients’ needs. A nurse reflected upon ethical dilemmas that she usually discusses with peers:

*We’re good at highlighting ethical problems with technology. We rarely discuss more hidden problems, but instead conclude that whenever we determine what’s ethically right we’re on the right track.*

Some participants reported that the technology improved their relationship with patients, particularly by allowing them to care for patients without having to focus on medication. For some patients, digital medicine dispensers made administration of medication more predictable than other arrangements had and participants expressed that if patients perceived the benefit of using technology to receive higher quality of care, then patients were likely to be more comfortable with the surveillance that accompanied using the technology. In one instance, the ideal of delivering so-called ‘warm care’ was challenged by a story about a patient who had reportedly said ‘There’s nothing wrong with the focus on warm hands, but for me, it’s more important to get my medicine at the right time’. To encourage less willing patients to accept the technology, participants reported giving them information about the benefits of using the devices. However, it remained unclear whether patients were made aware of possibilities for monitoring their lifestyles. As participants indicated, as long as healthcare professionals felt that patients could be helped even by means of additional surveillance possibilities, then implementing technology was not portrayed as a problem:

*We thought they would be able to manage better and take their own medicine even if they were allowed to use the medicine dispenser. Because in that way, we would be able to control whether they took their medicines* (Assistant nurse).

Among other unexpected consequences of using the technology participants reported, was that even if patients managed to become independent in administrating their medication by using the digital medicine dispenser, a new form of dependence for patients and their next-of-kin emerged when technical problems occurred and they required assistance from healthcare professionals. Although participants claimed that such threats to autonomy sparked distrust in new services and technologies among patients, they also perceived that problems with the technology typically stemmed from user error. As one nurse explained:

*You should remember that this is technology and that it’s not infallible. You should avoid for instance using GPS technology or a medicine dispenser alert system as an excuse for doing nothing as long as it doesn’t sound an alarm, because one day it will suddenly fail. Its battery could go dead without anyone noticing, or something else could go wrong. In that sense, technology can never replace human contact. An alarm won’t go off every time a user has a fall injury or fails to take his or her medicine.*

Altogether, implementing technology such as digital medicine dispensers introduced new, unforeseen opportunities and challenges for the relationship between home healthcare professionals and their patients.

## Discussion

The findings indicate that introducing new technology in home healthcare services requires healthcare professionals’ personal justification and rationale for such action, because their opinions and approval influenced whether they welcomed digital medical dispensers or not. Also crucial was coherence between the technological mechanisms of medicine dispensers and the perceived care needs of patients. Among other findings, relationships with patients could be improved if healthcare professionals concluded that new technology improved quality of care. Lastly, although participants often gave voice to well-recognized ethical implications of using new technology, including replacing face-to-face care with impersonal technology, they did not discuss more obscure consequences such as access to unnecessary data about patients due to using the technology.

More particularly, the findings suggest that pressure from national and local authorities could heavily influence how healthcare professionals perceive the need for new technology. Among the home healthcare professionals in our sample, pressure to make time and resources available posed implications for their relationships with patients. They had to solve problems caused by time constraints and reduced health workforce by way of innovation, including the use of new technology. Unsuccessful implementation would have to be solved by the healthcare professionals alone. Although telecare and assistive technology have been proposed as solutions to new demands facing healthcare services, research has shown that the benefits of introducing a particular technology are not sufficient for solving challenges related to demographic changes and their demands for resources [37, 38]. Home healthcare professionals experience such challenges in their everyday work, due to lack of skilled professionals in home healthcare services and the unprecedentedly higher share of complex patient conditions [3]. Nevertheless, some healthcare professionals may be reluctant to use technology if it implies risking their relationships with patients [39].

However, because care that is more efficient can mean better care for example enhancing patients’ autonomy or delaying their institutionalisation, participants in our study expressed that implementing new technology could strengthen their relationships with patients. At the same time, by improving older and disabled persons’ likelihood of living at home for longer, healthcare services could achieve the dual goal of facilitating a more satisfying life for the patients and enhancing the effectiveness of services at the organisational level [7, 40, 41]. Of course, implementing new technology in home healthcare services requires a systematic strategy for service innovation in the organisation, and the responsibility for embracing the new technology should not fall to individual healthcare professionals and their patients [39].

A recurring concern among the home healthcare professionals interviewed was how digital medicine dispensers could support care that empowers patients and boosts self-care. The technology had enabled patients to take their medicine independently, while healthcare professionals could remotely monitor their intake and take action when problems arose. That result supports earlier findings that healthcare professionals are more positive about technology that enforces caring ideologies that support patient autonomy and empowerment [6, 40]. Telecare seems to make practice a policy of ageing-in-place by supporting the independence and wellbeing of patients [7, 42]. By contrast, our results indicate that healthcare professionals considered installing new technology in the homes of certain patients worsen their patient-caregiver relationships. Scepticism and resistance to remote monitoring of medicine intake could stem from the idea that healthcare is relational and can only be delivered in person [8, 28, 43–45]. Such a notion is especially significant when patients are exceptionally dependent, have cognitive difficulties or are old and frail [26, 46]. Health professionals interviewed in our study reported that introducing technology in those cases instead of empowering patients could make the patient even more dependent for instance on informal care. Furthermore, they expressed that it could also disempower patients who were uncomfortable with the technology or in need of a type of healthcare that demands human presence. Living independently with some assistance is a humanistic goal, since institutionalisation can be perceived as a threat to older and disabled people’s integrity [47]. In addition, home healthcare is more cost-effective than inpatient and long-term care in institutions, chiefly because the former can benefit from the service user’s relatives, volunteers and housing situation [47]. Therefore, efforts should be made to achieve the goal of more people ageing at home. In any case, technology should be conceived as a supplement to, not a replacement for, face-to-face contact, and the safety and appropriateness of replacing healthcare with technology warrant further discussion [44, 48, 49].

Findings also demonstrate that in some cases, digital medicine dispensers can provide home healthcare professionals and services with previously unknown information about patients, both medically relevant information and personal information beyond the scope of healthcare. Such previously inaccessible insights into patients’ lives, can jeopardise patients’ privacy at home [8, 23]. Most people concur that being monitored in one’s home or having movements detected represents an extreme intrusion into a person’s life [50]. Surprisingly, however, the healthcare professionals interviewed did not perceive those new possibilities as being ethical dilemmas and had not reflected on how they could change their understandings of and relations to patients. Moreover, the possibility raises questions about the acceptable level of surveillance in home healthcare services in exchange for other benefits, including patient empowerment and service efficiency. Such consequences need to be discussed individually regarding each patient in connection with the actual technology introduced [51].

### Strengths and limitations

A strength of the study was its multi-case design and multi-disciplinary focus, both of which furnished information about the introduction of digital medicine dispensers across different Norwegian municipalities and could increase the transferability of its findings. The research team consisted of researchers from diverse disciplines, including healthcare, design and sociology, which facilitated broad discussions and multiple perspectives on the interpretation of data and, in turn, strengthened the trustworthiness of findings. However, the study also presented some limitations. Regarding policy demands for telehealth and assistive technologies, we had expected digital technology for medication to be widely used in home healthcare services, whereas in reality, few municipalities had implemented such technology. This necessitated performing an initial inquiry to identify relevant cases and recruiting a purposive sample. Some participants were recruited through their managers, and municipalities selected as cases had a special focus on technology and eHealth, which might have caused an overrepresentation of professionals with a positive attitude toward digital medicine dispensers. Among municipalities, progress in testing and implementing technologies varied considerably, and some municipalities had performed only small-scale pilots of the devices. In early phases of implementation processes, healthcare professionals might be very enthusiastic, which can prompt great, even unrealistic expectations of positive effects. Lastly, we included only the perspective of healthcare professionals. Although we acknowledge the significance of patients’ perspectives when studying experiences of care, representing that perspective was beyond the scope of the study. We aimed to present healthcare professionals’ experiences as a starting point for research on experiences with eHealth and telecare in home healthcare services.

## Conclusions

Faced with demographic changes expected to imply increased demands for healthcare services, home healthcare services also face immense pressure to find innovative, efficient way of working in response. Technologies such as digital medicine dispensers with remote healthcare have been introduced to support a dual objective: offering service that is more efficient and enhancing patient independence in order to reduce demand. The present study has shown that when healthcare professionals perceive that technology is introduced in a manner that supports patient empowerment and maintains people’s trust in the service and service quality, technology can help to mitigate experienced challenges and even to achieve the goals. Relationships between professionals and patients, however, might be at stake if healthcare professionals conclude that technology does not cohere with the needs of individual patients or fails to facilitate safe, trustworthy service. Healthcare professionals need to carefully assess patients’ care needs and preferences, as well as align and adjust the introduction of telecare technology to those needs and preferences. Careful reflection on the benefits of technology compared to potential disadvantages and ethical implications is crucial.

## Abbreviations

ASE: Accredited Social Educator
GP: General Practitioner
GPS: Global Positioning System
ICT: Information and Communication Technology
NSD: Norwegian Centre for Research Data.
RN: Registered Nurse
